# 2121 Quantitative structural knee measurements improve classification of accelerated knee osteoarthritis: Data from the osteoarthritis initiative

**DOI:** 10.1017/cts.2018.112

**Published:** 2018-11-21

**Authors:** Lori L. Price, Timothy E. McAlindon, Mamta Amin, Charles B. Eaton, Julie E. Davis, Bing Lu, Grace H. Lo, Michael E. DeBakey, Jeffrey Duryea, Mary F. Barbe, Jeffrey B. Driban

**Affiliations:** 1 Tufts Medical Center; 2 Temple University School of Medicine; 3 Alpert Medical School of Brown University; 4 Brigham & Women’s Hospital and Harvard Medical School; 5 VAMC & Baylor College of Medicine

## Abstract

OBJECTIVES/SPECIFIC AIMS: The aim of this study is to determine whether quantitative measures of knee structures including effusion, bone marrow lesions, cartilage, and meniscal damage can improve upon an existing model of demographic and clinical characteristics to classify accelerated knee osteoarthritis (AKOA). METHODS/STUDY POPULATION: We conducted a case-control study using data from baseline and four annual follow-up visits from the osteoarthritis initiative. Participants had no radiographic knee osteoarthritis (KOA) at baseline. AKOA is defined as progressing from no KOA to advance-stage KOA in at least 1 knee within 48 months. AKOA knees were matched 1:1 based on sex to (1) participants who did not develop KOA within 48 months and (2) participants who developed KOA but not AKOA. Analyses were person based. Classification and regression tree analysis was used to determine the important variables and percent of variance explained. RESULTS/ANTICIPATED RESULTS: A previous classification and regression tree analysis found that age, BMI, serum glucose, and femorotibial angle explained 31% of the variability between those who did and did not develop AKOA. Including structural measurements as candidate variables yielded a model that included effusion, BMI, serum glucose, cruciate ligament degeneration and coronal slope and explained 39% of the variability. DISCUSSION/SIGNIFICANCE OF IMPACT: Knee structural measurements improve classification of participants who developed AKOA Versus those who did not. Further research is needed to better classify patients at risk for AKOA.

